# Antibacterial Activity of a Novel Oligosaccharide from *Streptomyces californics* against *Erwinia carotovora* subsp. *Carotovora*

**DOI:** 10.3390/molecules27082384

**Published:** 2022-04-07

**Authors:** Maysoon Abdulrahman Al-Zubairy, Khaled Hussein, Salwa H. Alkhyat, Abdullah Yahya Al-Mahdi, Saeed Munassar Alghalibi, Adel Ali Al-Gheethi, Muhanna Mohammed Al-Shaibani, Hesham Ali El Enshasy, Nik Marzuki Sidik

**Affiliations:** 1 Microbiology Section, Biological Sciences Department, Faculty of Sciences, Sana’a University, Sana’a 12544, Yemen; alkhyat11@gmail.com (S.H.A.); alghalibi@gmail.com (S.M.A.); 2 Chemistry Department, Faculty of Sciences, Sana’a University, Sana’a 12544, Yemen; drkhaled26@yahoo.com; 3 Department of Microbiology, Faculty of Medicine, Lincoln University College, Petaling Jaya 47301, Malaysia; drabdullahyahya@lincoln.edu.my; 4 Micro-Pollutant Research Centre (MPRC), Department of Civil Engineering, Faculty of Civil Engineering & Built Environment, Universiti Tun Hussein Onn Malaysia, Parit Raja, Batu Pahat 86400, Malaysia; muhanna@uthm.edu.my; 5 Institute of Bioproducts Development (IBD), Universiti Teknologi Malaysia (UTM), Skudai 81310, Malaysia; henshasy@ibd.utm.my or; 6 City of Scientific Research and Technology Applications (SRTA), New Burg Al Arab, Alexandria 21934, Egypt; 7 Faculty of Agro-Based Industry, Universiti Malaysia Kelantan, Jeli 17600, Malaysia

**Keywords:** antibacterial activity, optimization, oligosaccharide, *Streptomyces californics*

## Abstract

The present study aims to characterize and predict models for antibacterial activity of a novel oligosaccharide from *Streptomyces californics* against *Erwinia carotovora* subsp. *carotovora* using an adaptive neuro-fuzzy inference system and an artificial neural network. The mathematical predication models were used to determine the optimal conditions to produce oligosaccharide and determine the relationship between the factors (pH, temperature, and time). The characteristics of the purified antibacterial agent were determined using ultraviolet spectroscopy (UV/Vis), infrared spectroscopy (FT-IR), nuclear magnetic resonance spectroscopy (^1^H- and ^13^C-NMR)**,** and mass spectrometry (MS). The best performances for the model were 39.45 and 35.16 recorded at epoch 1 for *E. carotovora* Erw_5_ and *E. carotovora* EMCC 1687, respectively. The coefficient (R^2^) of the training was more than 0.90. The highest antimicrobial production was recorded after 9 days at 25 °C and a pH of 6.2, at which more than 17 mm of the inhibition zone was obtained. The mass spectrum of antimicrobial agent (peak at R.T. = 3.433 of fraction 6) recorded two molecular ion peaks at *m/z* = 703.70 and *m/z* = 338.30, corresponding to molecular weights of 703.70 and 338.30 g/mol, respectively. The two molecular ion peaks matched well with the molecular formulas C_29_H_53_NO_18_ and C_14_H_26_O_9_, respectively, which were obtained from the elemental analysis result. A novel oligosaccharide from *Streptomyces californics* with potential activity against *E. carotovora* EMCC 1687 and *E. carotovora* Erw_5_ was successfully isolated, purified, and characterized.

## 1. Introduction

*Streptomyces* are the group of Gram-positive filamentous bacteria growing in various natural environments and classified among the richest natural sources for antibacterial and antitumor activity, as well as antifungals, antivirals, anti-hypertensives, and immunosuppressives [1]. It has been reported that most of the medically useful antibiotics were produced by *Streptomyces* spp. [2]. The novel antimicrobial products from *Streptomyces* spp. have received high attention in recent years due to the increasing antimicrobial resistance among the pathogenic bacteria toward currently used antibiotics such as chloramphenicol, tetracycline, macrolide, and vancomycin, which was also isolated from diverse *Streptomyces* spp. [3]. The new trends in the antimicrobial activity research looking to find novel compounds with high activity against the infectious agents but with a mechanism differ from those reported for currently used antibiotics. That aside, researchers have focused mainly on developing a prediction model to expect the time required to develop resistance mechanisms among the pathogenic bacteria against the new antimicrobial compounds from *Streptomyces* spp.

The application of antimicrobial compounds from *Streptomyces* spp. as a biological control is a very promising alternative to pesticides. Studies have detected undesirable chemical residues in the food chain with adverse effects on human health [4]. The utilization of natural antagonism between microorganisms to protect plants has been reported in several studies [5]. *Erwinia carotovora* subsp. *carotovora* is one of the most destructive plant pathogen bacteria [6], as the bacterium strain causes bacterial soft rot in potatoes and other crops [7]. It is the major pathogen affecting potato seed tuber pieces after cultivation, during vegetative growth, and in the storage period [8]. *Erwinia* sp. is a heterologous group of Gram-negative, rod-shaped motiles with peritrichous flagella, non-sporing, and facultative anaerobic pectolytic plant pathogens [9]. The bacterium species are classified in *Pectobacterium* as *P. carotovorum* subsp. *carotovorum*, *P. carotovorum* subsp. *atrosepticum*, *P. chrysanthemi* “*Dickeya* spp.” [10,11], *Bacillus thuringiensis*, *B. cereus*, *B. subtilis*, *B. megaterium*, *B. pumilus* [12], *Cyanodermella* sp. [13], *Paenobacillus polymyxa* [14], *B. subtilis, P. fluorescens*, *Rhizobium leguminosarum, T. harzianum*, *A. flavus* [15], *P. fluorescens, B. subtilis*, *B. thuringiensis* [16], and *Rhizopus stolonifer* [17]. Baz et al. [18] revealed that *Streptomyces* sp. OE7 showed the potential to control soft rot on potato slices. However, studies on the utilization of biological control agents active against blackleg and soft rot bacteria are still at the lab scale. The main limitation lies in the requirement for the antagonist to satisfy several criteria [19]. The technology transfer from small-scale to large-scale field testing is difficult due to the annual variations in the weather, resulting in a lack of consistency in the results. The application of biological control strains should consider the environmental stresses affecting microbial activities. Therefore, the exploring an effective indigenous agent is one of the most effective solutions [20]. 

The inactivation mechanism of the antimicrobial agents from the biocontrol microorganisms belongs to alkaloids, elimoklavine, festuklavine, agroklavine, ergometrine, bacteriocins, and iturins [21,22,23]. Cladera-Olivera et al. [24] reported that the bacteriocin-like substance produced by *B. licheniformis* exhibited bactericidal activity against *E. carotovora* subsp. carotovora. The lethal mechanisms act by interacting with cell membrane lipids, provoking lysis of the cells.

The adaptive neuro-fuzzy inference system (ANFIS) and artificial neural network (ANN) are among the advanced and powerful modeling tools used in medical and environmental applications. Nevertheless, very limited studies utilized mathematical predication modeling for investigating the production of antimicrobial agents from natural sources. These models are used to simulate the behaviors of antimicrobial substance activities toward infectious agents as a response to the production parameters at a large scale, since these models provide accurate predictions for the degradation process of antibiotics [25].

The current work aims to characterize a new antimicrobial compound from *Streptomyces* sp. and its biological control activity against *E. carotovora* subsp. *Carotovora*.

The mathematical predication models, which include an ANFIS and ANN, are employed to determine the optimal conditions for antimicrobial substance production and activation against *E. carotovora* subsp. *Carotovora* and determine the interactions between the factors (pH, temperature, and time). These models provide more details on the nature of the production process and reveal the response of *E. carotovora* subsp. *Carotovora* to the activity of antimicrobial substances in different conditions.

## 2. Results and Discussion

### 2.1. Optimization and Prediction Models Using ANFIS and ANN Simulations

**Figure 1:**
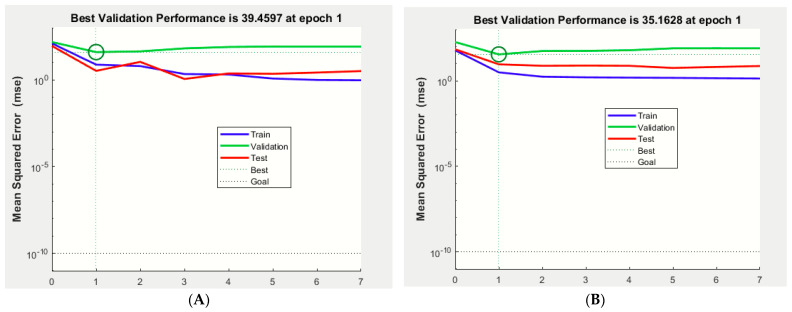
Best validation performance for ANFIS analysis with low MSE and minimal training RMSE was 0.365148. (**A**) *E.* carotovora Erw_5_. (**B**) *E. carotovora* EMCC 1687.

**Figure 2:**
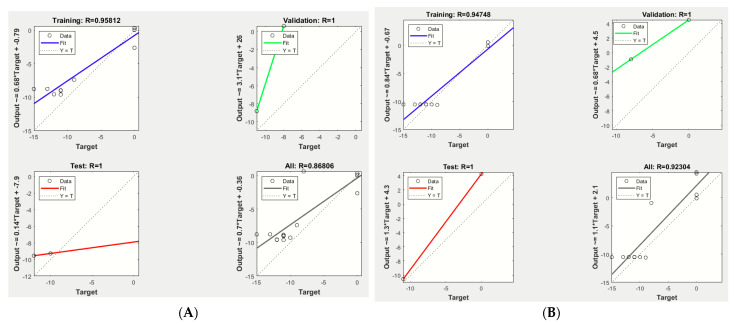
Coefficient (R^2^) of training, testing, and validation data for antimicrobial production as determined by ANN analysis. (**A**) *E.* carotovora Erw_5_. (**B**) *E. carotovora* EMCC 1687.

**Figure 3:**
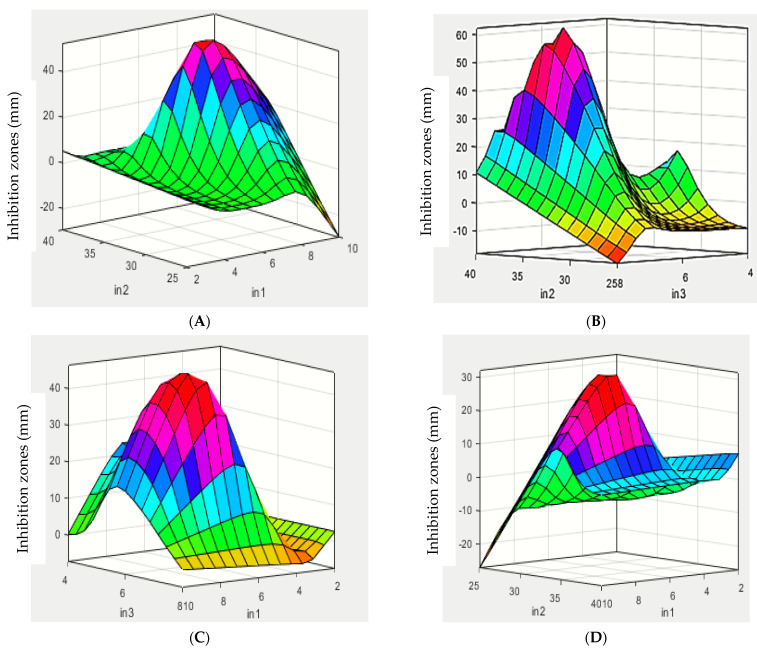

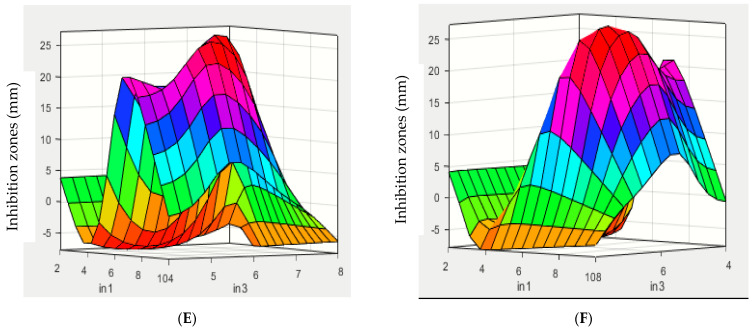
Antimicrobial agent production by *Streptomyces californics* as response to independent factors and predicted using ANFIS (time, day (In1), temperature (°C, In2), and pH (In3). (**A**–**C**) *E. carotovora* Erw_5_. (**D**–**F**) *E. carotovora* EMCC 1687.

The mathematical prediction model simulations were performed using an ANFIS with 7 epochs, and the results revealed that the best validation performances were 39.45 and 35.16, recorded at epoch 1 for *E. carotovora* Ewr_5_ and *E. carotovora* subsp. *carotovora* EMCC 1687, respectively, at which point the training, validation, and testing data exhibited similar mean square errors (MSEs) (Figure 1). The MSE is a statistical indicator for estimating the average of the squares of the errors [26]. The low values indicate the model’s accuracy in the prediction of antimicrobial production. In this investigation, the lowest value of MSE was for training. The coefficient (R^2^) for training was 0.9581 vs. 0.9474 for *E. carotovora* Ewr_5_ and *E. carotovora* subsp. *carotovora* EMCC 1687, respectively, as detected using ANN analysis (Figure 2 and Figure 3), which indicated the close results between the actual experimental and predicted results, since the coefficient (R^2^) was more than 0.7 [27].

**Figure 4:**
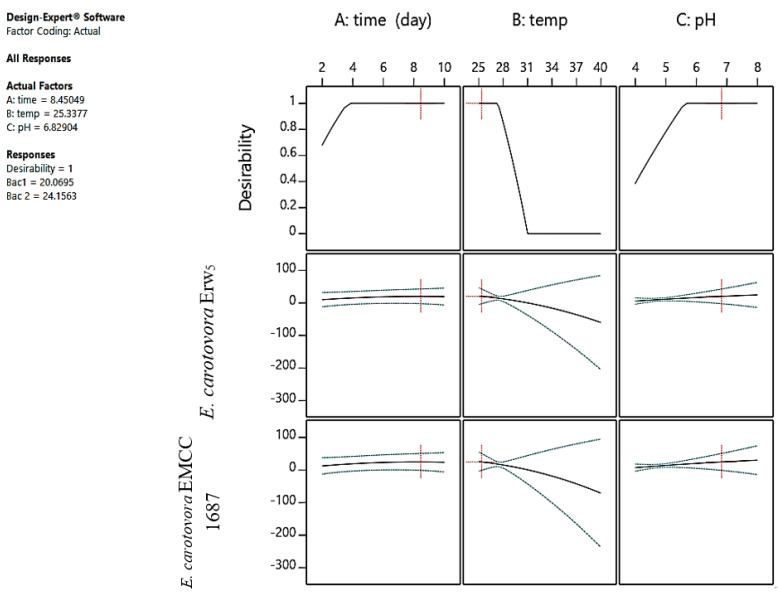
Best operating parameters for antimicrobial production by *S. californics* (22/30a) and antibacterial activity against *E. carotovora* Erw_5_ and *E. carotovora* EMCC 1687, as predicted by RSM analysis with central composite design (CCD).

The optimization of antimicrobial production by *S. californics* (22/30a) was performed using ANFIS with three independent factors, including temperature (25–40 °C), pH (4–8), and time (2–10 days) (Figure 4 and Figure S1). The results revealed that the production of antibacterial agents increased with the increasing incubation period, with diameter of inhibition zones of 11 and 13 mm against *E. carotovora* Ewr_5_ and *E. carotovora* EMCC 1687, respectively. The highest production of antibacterial agents was observed at 28 °C, with the highest inhibition zones being 13 and 15 mm against both *E. carotovora* Ewr_5_ and *E. carotovora* EMCC 1687, respectively. At 30 °C, the inhibition zones were 11 and 13 mm against both of *E. carotovora* Ewr_5_ and *E. carotovora* EMCC 1687, respectively, whereas no antibacterial activity was observed at the 25, 37, or 40 °C incubation temperatures. The effect of different initial pH values of the fermentation medium showed that the antibacterial activity increased with the decrease in pH, with the highest inhibition zones being 15 and 17 mm against both *E. carotovora* Ewr_5_ and *E. carotovora* EMCC 1687, respectively.

The effects of different carbon and nitrogen sources on the antibacterial production and activity against *E. carotovora* Ewr_5_ and *E. carotovora* EMCC 1687 are presented in Figure S2. It was noted that the maximum antibacterial inhibition against both strains occurred when the cultures were supplied with starch as a carbon source (12 vs. 14 mm for the inhibition zones in *E. carotovora* Ewr_5_ and *E. carotovora* EMCC 1687, respectively). On the other hand, the maximum antibacterial activity effect was observed when the culture was provided with KNO_3_ as a nitrogen source. An assay on the antibacterial activity of fractions of *S. californics* (22/30a) against *E. carotovora* Erw_5_ showed inhibition zones of 12 mm and 14 mm for *E. carotovora* EMCC 1687, as presented in Figure S3.

The results revealed that time exhibited more influence compared with the temperature, which had more of an effect than the pH level. In contrast, the pH exhibited more influence than time on the production of antimicrobial agents. The temperature and time as well as time and pH exhibited non-significant (*p* > 0.05) positive interactions, while temperature and pH had a non-significant negative interaction (*p* < 0.05). The first and quadratic models for the interactions between these factors are presented in Equations (1) and (2) with coefficient R^2^ being 0.7528 and 0.7614 for *E. carotovora* Ewr_5_ and *E. carotovora* EMCC 1687, respectively:

(1) $$y_{1}=-2.39+4.8x_{1}-29.79x_{2}-15.42x_{3}-26x_{1}x_{3}-4.0x_{1}^{2}-12.17x_{2}^{2}-1.4x_{3}^{2}$$

(2) $$y_{2}=-2.62+5.6x_{1}-35.36x_{2}-18.24x_{3}-31.01x_{1}x_{3}-5.14x_{1}^{2}-14.16x_{2}^{2}$$

The best operating parameters for antimicrobial production were predicted using ANFIS, with the highest antimicrobial production recorded after 9 days at 25 °C and a pH of 6.2, at which 17.8 and 21.34 mm inhibition zones against *E. carotovora* Ewr_5_ and *E. carotovora* EMCC 1687 were obtained (Figure 4).

The potential of *Streptomyces* spp. as a biological control against soilborne bacteria has been reported because of secondary metabolite production with the bioactivity [27]. However, secondary metabolite production relies on internal factors, such as carbon and nitrogen sources, and external factors, such as time, temperature, and pH level [28]. The production of secondary metabolites is affected by the availability of nutrients [29,30,31,32]. The maximum production of antimicrobial metabolite by *Streptomyces* sp. MNK7 was recorded after 10 days [33,34]. However, Singh and Rai [35] claimed that antibiotic production by *S. rimosus* MTCC 10792 was recorded after 24 hrs. These differences might be related to the microbe strain and the source of isolation. The best production of antimicrobial compounds by *Streptomyces* sp. is obtained at 28 °C [35], 30 °C [32], or 35 °C [33]. The highest production of antimicrobial agents from *Streptomyces* sp. was reported at pH levels of 5 [33], 7.0 and 7.5 [32,36], and 8 [37]. *S. griseocarneus* produces antimicrobial activity with glucose as a carbon source [34,38]. However, a quickly metabolized substance like carbohydrates enhances the production of antimicrobial production [39]. Nitrate, soybean meal, and peptone are among the best nitrogen sources for antimicrobial agent productions [29,36].

The antibacterial agent extracted from *S. californics* 22/30a was soluble only in water, DMSO, and hot methanol. This result agreed with Zamanian et al. [28], who reported that the active substance of *S. plicatus* (Strain 101) was water soluble and insoluble in chloroform, hexane, and dichloromethane. Kang et al. [29] found that the final dry extract from *Streptomyces* sp. strain JJ45 was soluble in water and insoluble in alcohol or ethyl acetate.

### 2.2. Characteristics of Antibacterial Agent from S. californics (22/30a)

The maximum activity of antibacterial extract from *S. californics* (22/30a) against *E. carotovora* Erw_5_ (A) and *E. carotovora* EMCC 1687 was recorded at a pH of 3 with inhibition zones of 19 and 22 mm, respectively (Figure S3). The antibacterial filtrate of *S. californics* (22/30a) exhibited stabile activity at room and boiling temperatures with inhibition zones of 12 and 14 mm, respectively (Figure S3). The antibacterial activity of crude extract was the highest, with inhibition zone diameters of 19 and 20 mm against *E. carotovora* Erw_5_ (A) and *E. carotovora* EMCC 1687, respectively. The crude antibacterial agent of *S. californics* (22/30a) had a light brown color, gummy nature, aromatic odor, and solubility in water, DMSO, and hot methanol, with 134–141 °C as the melting point.

The crude antibacterial extract was analyzed using TLC with different eluting systems to determine the suitable mobile phase for column chromatography (CC). Acetone-methanol-water (4:6:3) was the best eluting system which showed the presence of at least two different components of the crude antibacterial agent of *S. californics* (22/30a). Based on the column chromatography analysis, among 78 fractions, only 6 fractions (41, 43, 44, 45, 47, and 50) showed antibacterial activity against *E. carotovora* Erw_5_ and *E. carotovora* EMCC 1687, with an inhibition zone of 24 mm against *E. carotovora* EMCC 1687 in the case of fraction 47 and 19 mm against Erw_5_ in the case of fraction 44 (Figure S3). All fractions were analyzed using TLC and showed that all fractions were like each other and consisted of two components. The fractions with antibacterial activity against both *E. carotovora* Erw_5_ and *E. carotovora* EMCC 1687 were divided into six fractions using the fractional crystallization method. Only two fractions (4 and 6) showed antibacterial activity against both of Erw_5_ and *E. carotovora* subsp. *carotovora* EMCC 1687 (Figure S3). Fraction 6 of the antibacterial agent was chosen to be identified using certain chemical reagents and spectroscopic analysis.

To identify the chemical type of the antimicrobial agent, qualitative analysis was carried out using certain chemical testes (Molisch, ninehydrine, and sodium nitrobrocide tests). The reaction of the antimicrobial agent with the sodium nitrobrocide reagent returned a negative result, whereas the reaction of the antimicrobial agent with ninehydrine reagents gave positive results. With the Molisch reagent, and before carrying out any chemical treatments for the antimicrobial agent, this fraction did not give a positive result, while afterward it was broken down into monosaccharides by acid, giving a positive test result. These results reveal that the antimicrobial agent contained nitrogen atoms in its structure which belonged to carbohydrate compounds.

**Figure 5:**
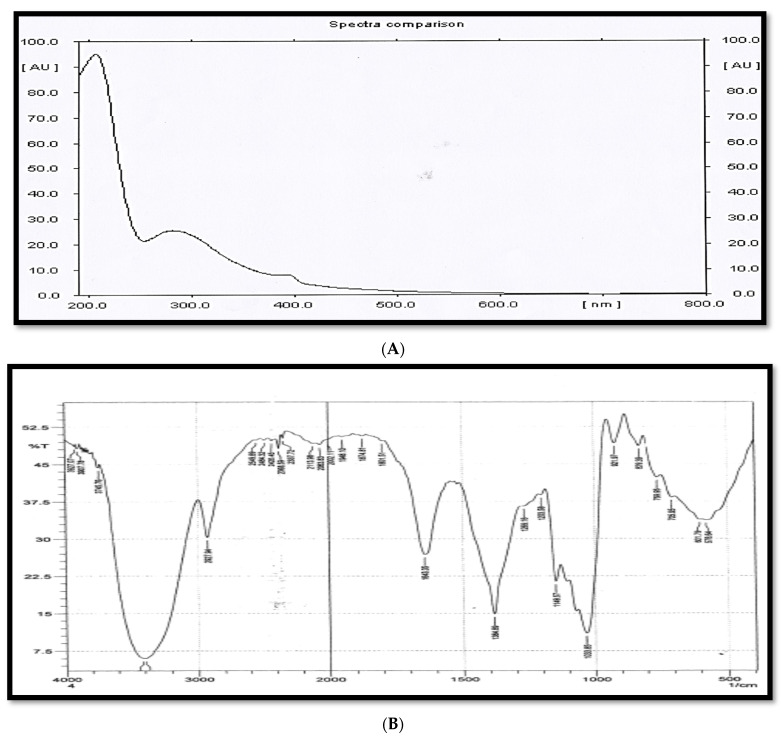

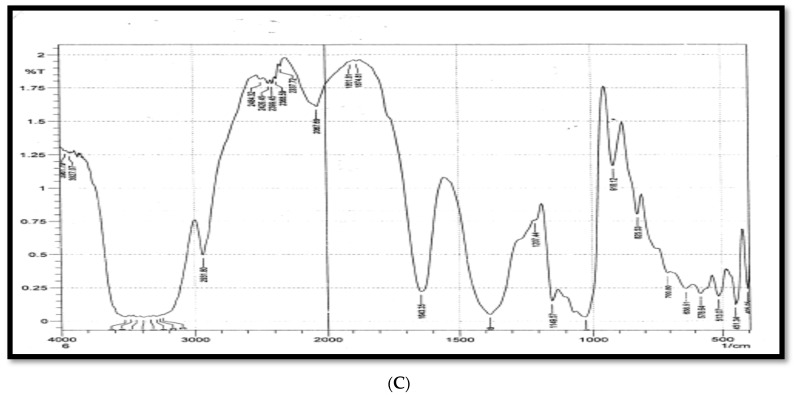
(**A**) UV/Vis spectrum. (**B**) The FTIR spectrum of the compound. (**C**) FTIR spectrum of fraction 6.

The UV/Vis spectrum of the purified component showed that the spectrum of the pure compound was determined in the region (200–400 nm) by using methanol as a solvent. The UV/Vis spectrum recorded a maximum absorption band at λ_max_ 210 and 279 nm (Figure 5A). The compound in its IR spectrum exhibited bands at 3670–3050, 2931, 1643, 1384, 1269, 1207, 1149, 1033, 918, and 825–513 cm^−1^, from which the presence of a primary amine, hydroxyl groups, and glycosides bond bands were inferred (Figure 5B).

The UV/Vis and FT-IR spectral data of C_29_H_53_NO_18_ were in complete agreement with those reported in the literature for oligosaccharides and polysaccharides [37,38,39,40,41,42,43].

The presence of a broad and distorted absorption band in the range of 3670–3050 cm^−1^ in the IR spectrum of the antibacterial agent was due to overlap between the absorption bands of alcoholic O-H and N-H of the primary amine’s stretching vibration, whereas the absorption band at 2931 cm^−1^ was due to sp^3^ C-H stretching vibration.

An important indication of this interference was the disappearance of only this distortion in the infrared spectrum of component 4 (ret. time: 3.350) of fraction 6 (Figure 5C), which did not contain in its structure the amino group (NH_2_). In addition, the appearance of the strongest absorption band at 1643 cm^−1^ in the IR spectrum of component 6 (Figure 5B) was due to the N-H bending vibration of the primary amine [44], whereas the presence of a moderate (less intense) absorption band at the same position (1643 cm^−1^) in the IR spectrum of component 4 (Figure 5C) was related to absorbed water [40].

The occurrence of a slightly broad absorption band at 1384 cm^−1^ corresponded to overlapping between the absorption bands of the C-N stretching vibration of the amine and sp^3^ C-H bending vibration, as well as the O-H bending vibration. The presence of ether (pyran and furan rings and methoxy) and glycoside bonds in the chemical structure of component 6 was confirmed by the appearance of a group of absorption bands at 1269, 1207, 1149, and 1033 cm^−1^, which corresponded to the stretching vibration of the C-O ether and C-O-C acetal bonds. The absorption bands at 918 and 709 cm^−1^ were due to O-H and N-H out-of-plane (oop) bending vibrations, respectively. The absorption band at 825 cm^−1^ indicates that the connection between the oligosaccharide units was β-glycosidic bonds [40]. The appearance of an absorption band at 578 cm^−1^ was attributed to a pyran-type sugar ring.

The results of the GC analysis of fraction 6 are shown in a GC chromatogram (Figure S4), which shows two overlapping peaks in retention time at 3.350 and 3.433 min. The mass spectrum of the antibacterial agent (peak at R.T. = 3.433 for fraction 6) recorded two molecular ion peaks at *m/z* = 703.70 and *m/z* = 338.30, corresponding to molecular weights of 703.70 and 338.30 g/mol (Figure S4B). The two molecular ion peaks matched well with the molecular formulae C_29_H_53_NO_18_ and C_14_H_26_O_9_, respectively, obtained from the elemental analysis. The mass errors for the two compounds were calculated based on the isotopic atomic masses of the elements and not based on their relative atomic masses.

**Figure 6:**
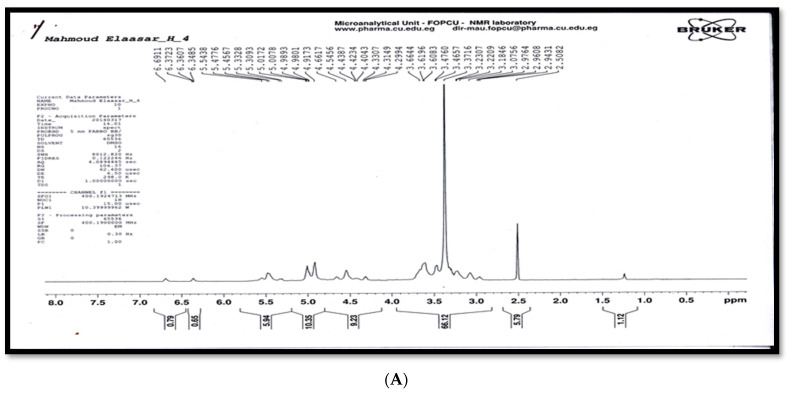

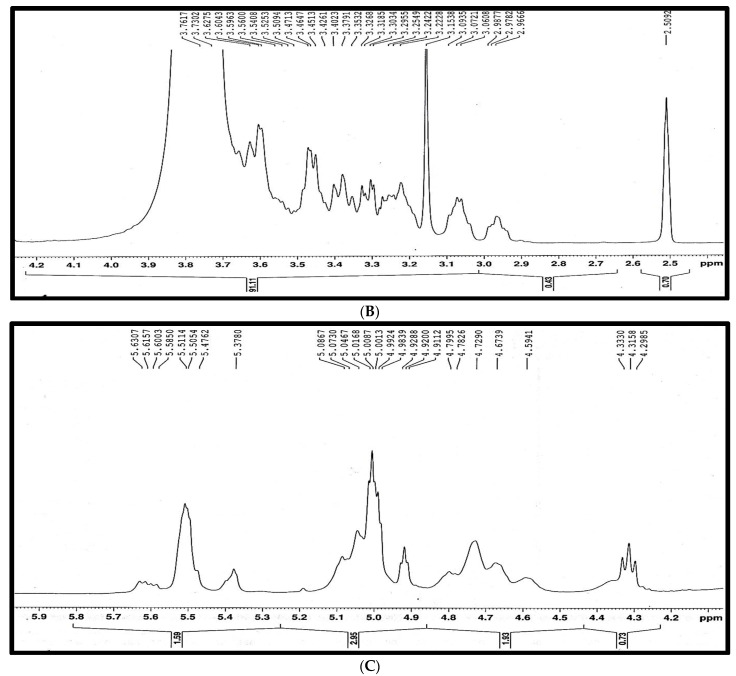
^1^H-NMR spectrum of the antibacterial agent (fraction 6), (**A**) Full spectrum, (**B**) Expansion of ^1^H-NMR spectrum partially from 2.00 ppm to 4.00 ppm, (**C**) Expansion of ^1^H-NMR spectrum partially from 4.20 ppm to 5.80 ppm.

**Figure 7:**
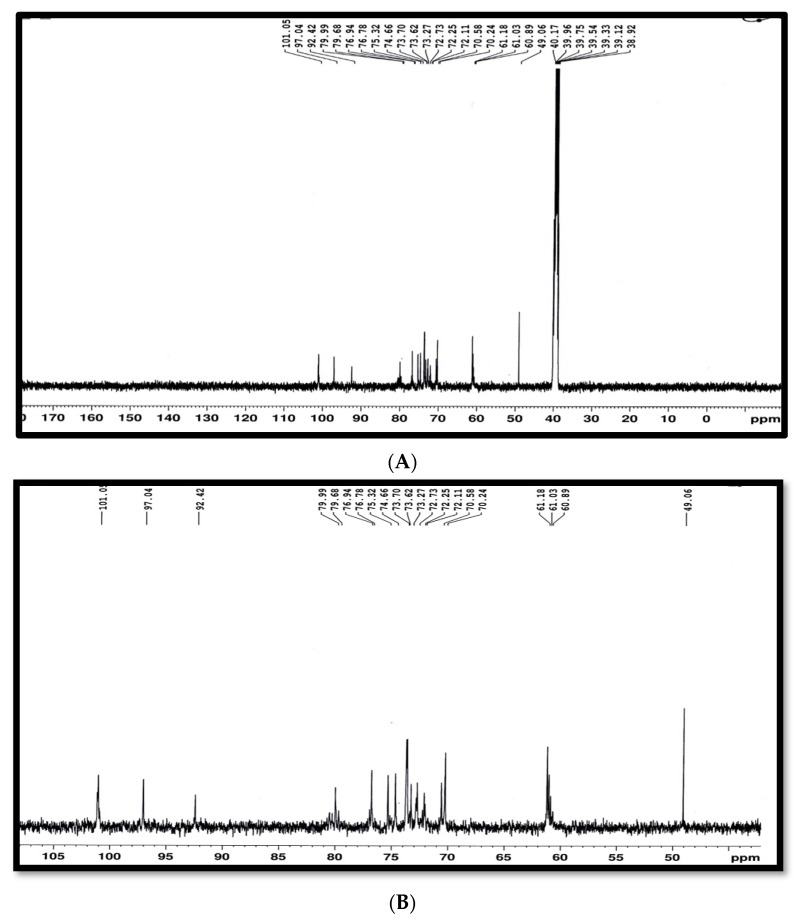
^13^C-NMR spectrum of the antibacterial agent (fraction 6), (**A**) showing full spectrum. (**B**) expansion of ^13^C-NMR spectrum partially from 45.00 ppm to 105.00 ppm.

**Figure 8:**
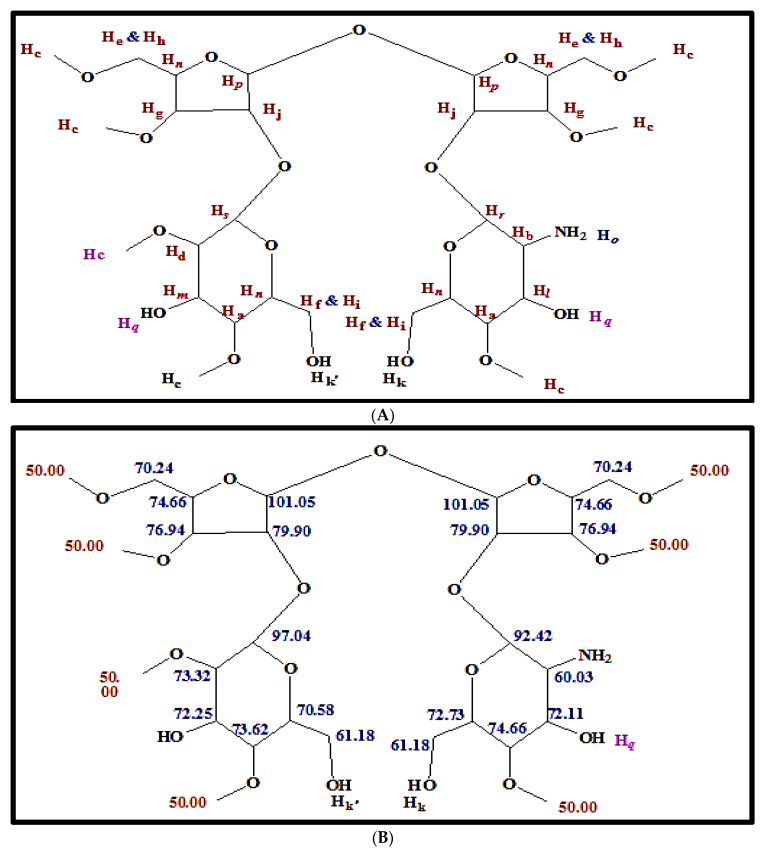
(**A**) Chemical structure of antibacterial agent (fraction 6) and chemical shift values of hydrogen (^1^H-NMR) by letters. (**B**) Chemical shift values of the carbon atoms (^13^C-NMR) displayed in antibacterial compound.

The different types of hydrogen and carbon were recorded from the signals in the ^1^HNMR and ^13^CNMR spectra of antibacterial agents, respectively, and matched well with the chemical structure in Figure 6A–C. However, the ^1^HNMR spectrum (400 MHz, DMSO) δ (ppm) (Figure 6A–C) exhibited signals at 2.9782 and 2.95818 (2 H, dd, *J* = 3.6 and 6.76 Hz, Ha), 3.0828 and 3.0529 (2H, dd, *J* = 8.56 and 6.32 Hz, Hb), 3.1538 (21H, s, Hc [O-CH_3_]), 3.18885 and 3.16875 (1H, dd, *J* = 6.12 and 3.56 Hz, Hd), 3.2509 and 3.21515 (2H, dd, *J* = 3.2 and 6.12 Hz, He), 3.3711 and 3.29325 (2H, dd, *J* = 6.4 and 3.32 Hz, Hf), 3.3942 and 3.29945 (2H, dd, *J* = 6.44 and 3.16 Hz Hg), 3.468 and 3.44365 (2H, dd, *J* = 3.64 and 6.12 Hz, Hh), 3.61975 and 3.6003 (2H, dd, *J* = 6.2 and 3.2 Hz Hi), 4.3158–4.2985 and 4.3330–4.3158 (2H, t, J = 6.39 and 6.68 Hz, Hj), 4.5941 (1H, br., s. Hk [OH]), 4.79105 and 4.6632 (1H, dd, *J* = 6.7 and 8.5 Hz, Hl), 4.7290 (1H, br., s. Hk’ [OH]), 4.9200 (1H, t, *J* = 3.52 Hz, Hm), 5.0013 (4H, m, Hn), 5.3780 (2H, br., s, Ho [NH_2_), 5.4762–5.4606 (2H, d, *J* = 6.24 Hz, Hp), 5.5114 (2H, br., s, Hq [2OH]), 5.6003–5.5850 (1H, d, *J* = 6.13 Hz, Hr), and 5.6307–5.6157 (1H, d, *J* = 6 Hz, Hs). The ^13^CNMR spectrum (100.63 MHz, DMSO) δ (ppm) exhibited signals as described in the chemical structure below and a distribution of carbon atoms in the compound. Further investigation of the chemical structure of the active component was carried out using ^1^HNMR and ^13^CNMR (Figure 6 and Figure 7, respectively) and matched well with the chemical structure in Figure 8. These findings agree with Kang et al. [29], who isolated α-L-sorbofuranose (3→2)-β-D-altrofuranose from *Streptomyces* sp. strain JJ45 with antibacterial activity. Many antibiotics consist of glycosides since the glycosidic residues are crucial for antibiotic activity. The glycosylation also improves the pharmacokinetic parameters of the antibiotics [44]. Carbohydrate-based antibiotics have received much attention in recent years as a new approach for antibiotic resistance [45]. Therefore, dimeric aminoglycosides represent best the candidates for carbohydrate-based antibiotics [46].

## 3. Materials and Methods

### 3.1. Streptomyces sp. Strain

The *Streptomyces* sp. were isolated from soil samples collected from Yemen according to the method described by Johnson and Curel [44]. The morphological characteristics of the *Streptomyces* spp. strain grown on starch casein agar medium (SCA) were described after a 10-day incubation period at 28 °C. The molecular analysis was conducted at The Regional Centre for Mycology and Biotechnology (RCMB) at Al Azhar University (Egypt). The strain was identified as *Streptomyces californics.*

### 3.2. Test Bacterial Strain

*E. carotovora* subsp. *carotovora* EMCC 1687 was used as a control, while *E. carotovora* subsp. *Carotovora,* a local strain (*E. carotovora* Erw_5_), was isolated from a potato tuber infected with soft rot as described by Johnson [45]. The bacterial strain was identified based on the morphological characteristics.

### 3.3. Production of Antibacterial Agent from Streptomyces sp. Strain

The production of an antibacterial agent from *S. californics* 22/30a was conducted in a starch nitrate broth medium containing (g L^−1^) 20.0 of KNO_3_, 20.0 of K_2_HPO_4_, 1.0 of KCl, 0.5 of MgSO_4_, 0.5 of FeSO_4_, and 0.01 and 2 of CaCO_3_. The pH was adjusted to 4 using 0.1 N of HCl. The inoculated media (100 mL) with *S. californics* 22/30a was incubated on a rotary shaker incubator (200 rpm) at 28 °C for 10 days. The culture broth was subjected to centrifugation at 4000 rpm for 15 min, and 100 µL of supernatants was transferred into a well of 7 mm made in NA plates seeded with target *E. carotovora* strains. The plates were kept in a refrigerator for 2 h and then incubated at 28 °C for 24 h. The antibacterial activity was recorded in terms of the inhibition zone of the target *E. carotovora* strains around the well of the supernatant [46].

### 3.4. Adaptive Neuro-Fuzzy Inference System (ANFIS) and Artificial Neural Network (ANN) Analysis

**Figure 9:**
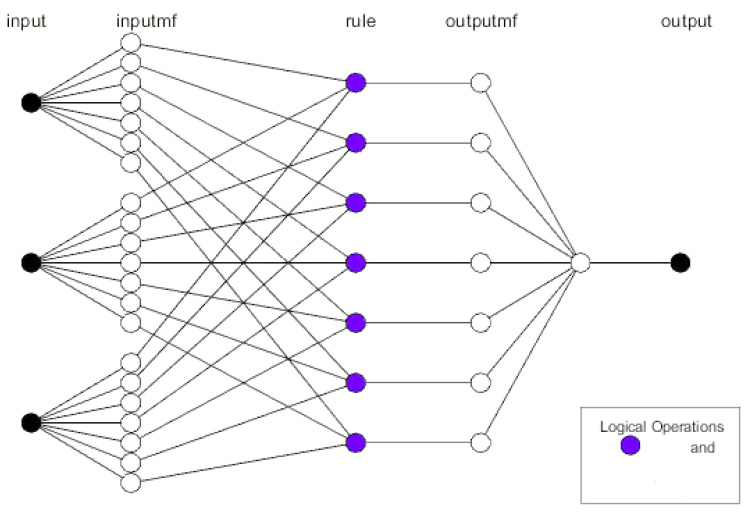
The input layer (time, day (In1), temperature (°C, In2) and pH (In3)) and output layer (inhibition zone of *Erwinia carotovora*) used in the adaptive neuro-fuzzy inference system (ANFIS) and artificial neural network (ANN) analysis.

The ANFIS and ANN models were used in the current work to investigate the production of antimicrobial agents from *S. californics* 22/30a as a response to the temperature (25–40 °C), pH (4–8), and time (2–10 days). The developed models consisted of three layers, as shown in Figure 1. The input layer consisted of five neurons (parameters) which were represented by input 1 (time, day (In1), temperature (°C, In2), and pH (In3) (Figure 9)). The optimization process was conducted with the hybrid and 7 epochs to attain the best model. The proposed ANFIS and ANN model was executed in MATLAB R2021a. The experimental data were divided into training data (70%) and testing data (30%). The training, testing, and validation of the experimental data values were performed using an ANN and evaluated based on R^2^, while the MSE error was used to evaluate the proposed model’s performance.

### 3.5. Extraction, Separation, and Purification

The cell-free culture medium was mixed with ethanol (50:50 *v*/*v*) and centrifuged at 4000 rpm to remove the biomass residues. The supernatant was subjected to drying by evaporating the liquids using a dryer with a vacuum at 50 °C [47]. To test the activity of the crude extract, a fixed weight of the dry extract (0.1 g mL^−1^) was dissolved in distilled water, and 50 μL of the crude extract was assayed in triplicate as mentioned above (Section 3.1). The physical properties of the crude antibacterial agent (appearance, color, odor, and melting point) were determined using a Stuart Scientific Melting Point Apparatus (SMP3, UK).

A thin layer chromatography (TLC) aluminum plate of 20 × 20 cm coated with silica gel 60 F_254_ (Merck F254) was used to analyze the crude extract and to determine the suitable mobile phase for column chromatography (CC). The plate of TLC was developed at 25 ± 2 °C in a glass chamber saturated (*v*/*v*) with acetone-water (4:1), acetonitrile-water (4:1), methanol-water (4:1), and acetone-methanol-water (4:2:1, 4:5:3, 4:6:3, 6:2:3, 2:6:3, 2:4:4, 3:4:4, and 2:2:1) as mobile phases. The TLC plates were visualized under ultraviolet light at λ_254_ and λ_366_ nm [29,47].

The column chromatography (CC) loaded with silica gel (silica gel 60 with a 0.2–0.5-mm mesh) was used for a stepwise elution with five solvent systems in increasing order of polarity (in terms of volume ratio (*v*/*v*)). Seventy-eight fractions were obtained, and 2 (1–2), 24 (3–26), 24 (27–50), 24 (51–74), and 4 (75–78) fractions were eluted with pure acetone-methanol (from 6:0.5 to 3.5:3 (*v*/*v*)), acetone-methanol-water (from 3:3:0.5 to 0.5:3:3 (*v*/*v*)), methanol-water (from 3:3.5 to 0.5:6 (*v*/*v*)), and pure water in 20–25 mL for each fraction, respectively. The separated fractions (78 fractions) were concentrated and combined into 9 pools (1–9) based on TLC monitoring on silica gel 60 F_254_ using acetone-methanol-water (4:6:1 (*v*/*v*/*v*)) as a mobile phase. The TLC plates were visualized as mentioned above. The combined fractions (pool 1–9) were tested for their antibacterial activity against *E. carotovora* strains using the agar well diffusion method [28].

The purified combined fractions were subjected to further purification using fractional crystallization (FC) to 6 fractions at The Regional Centre for Mycology and Biotechnology (RCMB) at Al-Azhar University in Nasr City, Egypt. These fractions were screened via TLC using acetone-methanol-water (4:6:1 *v*/*v*/*v*) as the elution system to determine the purified fraction of the antibacterial agent. Two fractions were obtained and tested for their antibacterial activity against *E. carotovora* strains as described above (Section 3.3). Fraction number 6 was chosen for chemical characterization because of its activity against the pathogenic bacteria.

### 3.6. Characterization and Structural Elucidation of the Purified Antibacterial Agent

Spectroscopic analysis of the purified antibacterial agent was performed using ultraviolet spectroscopy (UV/Vis), infrared spectroscopy (FT-IR), and nuclear magnetic resonance spectroscopy (^1^H- and ^13^C-NMR), as well as mass spectrometry (EI-MS). The UV/Vis spectrum of the compound that was developed on a TLC plate (silica gel G-60 aluminum sheet, Merk, Darmstadt, Germany) was recorded using a CAMMAG TLC scanner system at the RCMB at Al-Azhar University in Egypt. The infrared spectra (FT-IR) were recorded using the potassium bromide disc technique with a Perkin-Elmer 1650 FT-IR spectrometer at the Microanalytical Center at Cairo University in Egypt.

The nuclear magnetic resonance (NMR) spectra were recorded on a Bruker high-performance digital FT-NMR spectrometer (^1^H 400 MHz; ^13^C 100.63 MHz) at 298.1 K using dimethylsulfoxide (DMSO) as the solvent and TMS as the internal reference, and the chemical shift was expressed in δ units (ppm) relative to the TMS. The coupling constants (*J*) were expressed in Hertz. The abbreviations used for multiplicities were *s = singlet, d = doublet, t = triplet, q = quartet,* and *m = multiplet*. All NMR experiments were conducted at the Microanalytical Unit (FOPCU) of the Faculty of Pharmacy at Cairo University in Cairo, Egypt (www.pharma.cu.edu.eg (accessed on 16 February 2022)). Mass spectral analysis was carried out on a direct Inlet part DI-50 to a mass analyzer by a Shimadzu GC/MS-QP5050 at the Regional Centre for Mycology and Biotechnology (RCMB), Al Azhar University, Egypt.

The gas chromatography of GC/MS was equipped with a 30 m × 0.25 mm (inside diameter) (*d_f_* = 0.25 μm) bonded phase DB-5 wax cross–linked fused silica capillary column covered with a film thickness of 0.5 μm of polydimethylsiloxane. The oven temperature was automatically increased from 30 °C for 3 minutes with a rate increment from 3 °C/min to 200 °C/min and isothermally held for 20 min at 200 °C. The linear helium carrier gas flow rate was fixed at 1 mL/min. The injector temperature was 140 °C, ion source temperature rest was 200 °C, and detector temperature was 200 °C. An Agilent model 6890 gas chromatograph interfaced with an Agilent 5791A mass selective detector (GC–MS) was used for mass spectral analysis of the GC components, and the mass spectrometer was an electron impact (EI) type with an MS ionization voltage of 70 electron volts, computerized from m/e 50 to m/e 800 (National Research Centre, Dokki, Cairo, Egypt).

## 4. Conclusions

A novel oligosaccharide from *Streptomyces californics* with activity against *E. carotovora* EMCC 1687 and *E. carotovora* Erw_5_ was successfully isolated, purified, and characterized. The optimization of the production process was successfully simulated and predicted using an ANFIS and ANN with R^2^ 0.9581 vs. 0.9474 for both bacterial strains, respectively. The best conditions for antimicrobial production and activity were obtained after 9 days at 25 °C and a pH of 6.2, at which there were 17.8- and 21.34-mm inhibition zones for *E. carotovora* Ewr_5_ and *E. carotovora* EMCC 1687, respectively. The mass spectrum determined two molecular ion peaks at *m/z* = 703.70 and *m/z* = 338.30, corresponding to molecular weights of 703.70 and 338.30 g/mol. The elemental analysis revealed that the molecular formulae of these compounds were C_29_H_53_NO_18_ and C_14_H_26_O_9_. The different types of hydrogen and carbon were determined by ^1^HNMR and ^13^CNMR. The findings of the present study indicate the efficiency of oligosaccharides, as carbohydrate-based antibiotics can use them as an alternative to glycoside-based antibiotics. The application of an ANFIS and ANN in the production simulation reflects the applicability of the production of antimicrobial agents on a large scale.

## Data Availability

Not applicable.

## Supplementary Materials

The following supporting information can be downloaded at: https://www.mdpi.com/article/10.3390/molecules27082384/s1. Figure S1: Effect of carbon and nitrogen sources at different temperatures on the antibacterial activity from *S. californics* (22/30a) against of *E. carotovora* Erw5 and *E. carotovora* EMCC 1687. (A) Carbon sources. (B) Nitrogen sources. Figure S2: Effects of different pH levels (A) and temperatures (B) of *S. californics* (22/30a) filtrate on antibacterial activity against *E. carotovora* Erw5 and *E. carotovora EMCC* 1687. Figure S3: Assay of antibacterial activity of fractions of *S. californics* (22/30a) against *E. carotovora* Erw5 and *E. carotovora* EMCC 1687. (A) Fractions 41 and 50. (B) *E.* fractions 4 and 6. Figure S4: GC chromatogram of fraction No. 6 (A) and mass spectrum of antibacterial agent fraction No. 6 (ret. time: 3.433) (B).

## Sample Availability

Not available.
