# Supplementary material for: Antibacterial Activity of a Novel Oligosaccharide from Streptomyces californics against Erwinia carotovora subsp. Carotovora
Source: Molecules. 2022 Apr 7;27(8):2384. doi: 10.3390/molecules27082384 (PMC9032947; doi:10.3390/molecules27082384)
Supplement: Supplementary file 1 [file molecules-27-02384-s001.zip › molecules-1622523-supplementary.pdf]

Antibacterial Activity of a Novel Oligosaccharide from *Streptomyces californics* against *Erwinia carotovora* subsp. *Carotovora*

Maysoon Abdulrahman Al-Zubairy [1,*], Khaled Hussein [2], Salwa H. Alkhyat [1], Abdullah Yahya Al-Mahdi [3], Saeed Munassar Alghalibi [1], Adel Ali Al-Gheethi [4,*], Muhanna Mohammed Al-Shaibani [4], Hesham Ali El Enshasy [5,6], Nik Marzuki Sidik [7,*]

[1] 1Microbiology Section, Biological Sciences Department, Faculty of Sciences, Sana'a University, Yemen

[2] Chemistry Department, Faculty of Sciences, Sana'a University, Yemen; drkhaled26@yahoo.com; alkhyat11@gmail.com; alghalibi@gmail.com

[3] Department of Microbiology, Faculty of Medicine, Lincoln University College, Selangor, Malaysia; drabdullahyahya@lincoln.edu.my

[4] Micro-Pollutant Research Centre (MPRC), Department of Civil Engineering, Faculty of Civil Engineering & Built Environment, Universiti Tun Hussein Onn Malaysia, 86400 Parit Raja, Batu Pahat, Johor, Malaysia

[5] Institute of Bioproducts Development (IBD), Universiti Teknologi Malaysia (UTM), Skudai 81310, Johor, Malaysia; henshasy@ibd.utm.my; hesham@utm.my

[6] City of Scientific Research and Technology Applications (SRTA), 21934 New Burg Al Arab, Alexandria, Egypt

[7] Faculty of Agro-Based Industry, Universiti Malaysia Kelantan, Jeli 17600, Kelantan, Malaysia

*** Correspondence:** adel@uthm.edu.my ; adelalghithi@gmail.com (AAG); alzubairymaysoon@yahoo.com (MAA); nikmarzuki@umk.edu.my (N.M.S.)

Supplementry data.

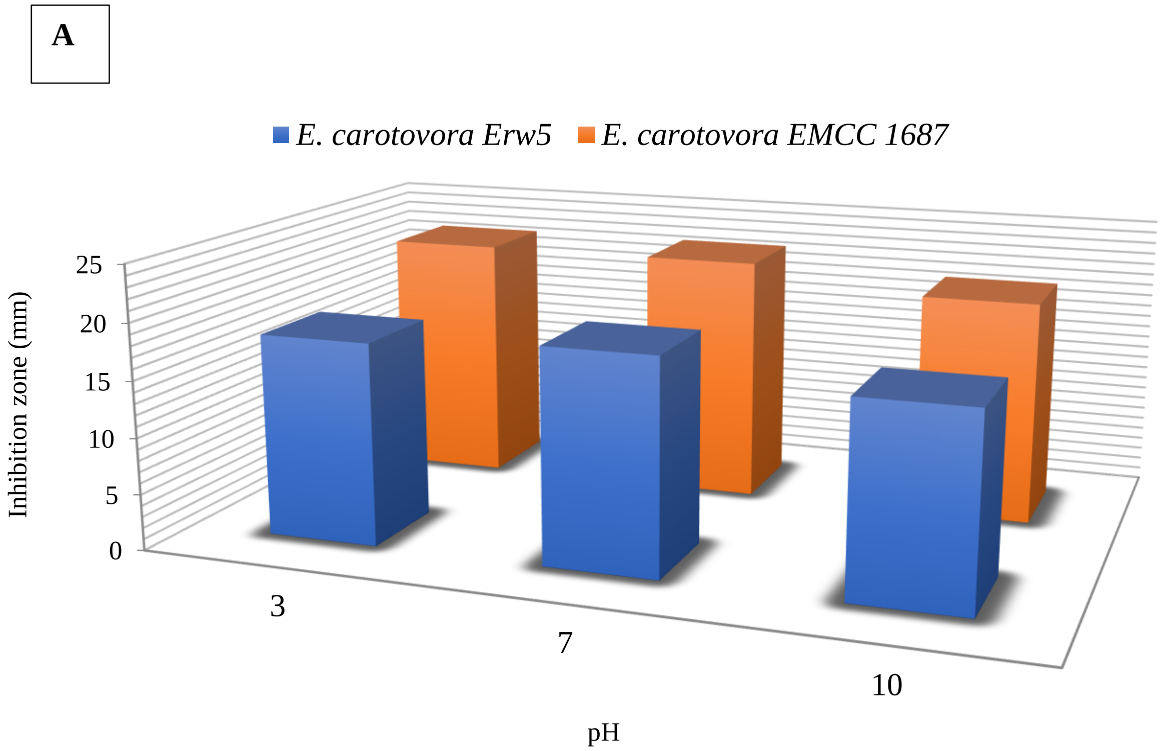

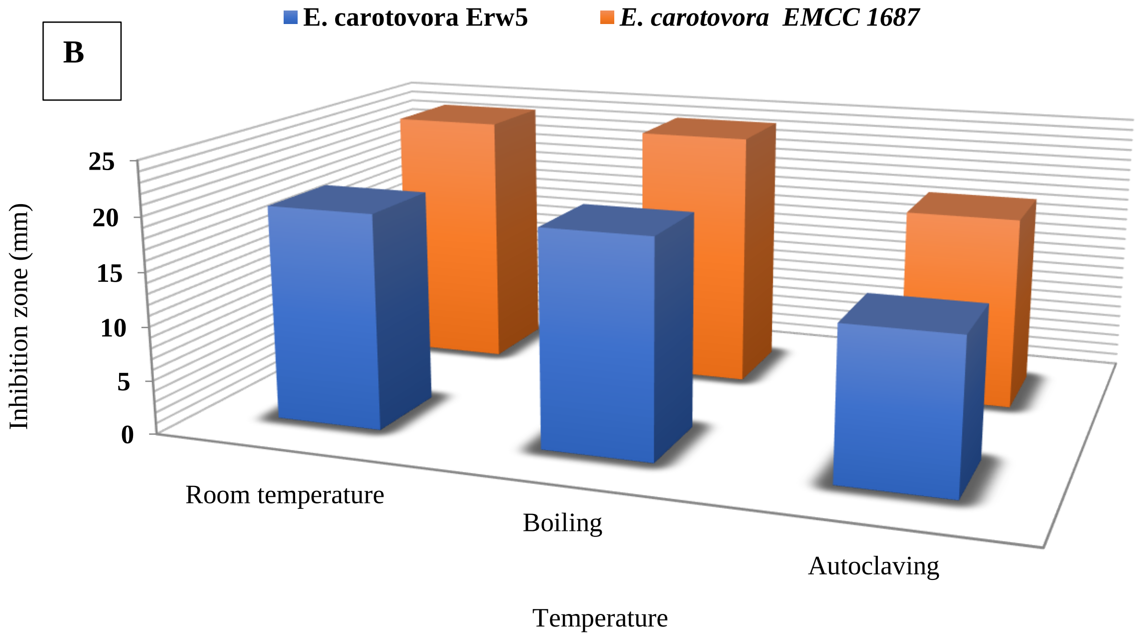

Figure S1: Effect different pH (A) and temperature (B) of *S. californics* (22/30a) filtrate on the antibacterial activity against *E. carotovora* Erw_5 and *E. carotovora* EMCC 1687.

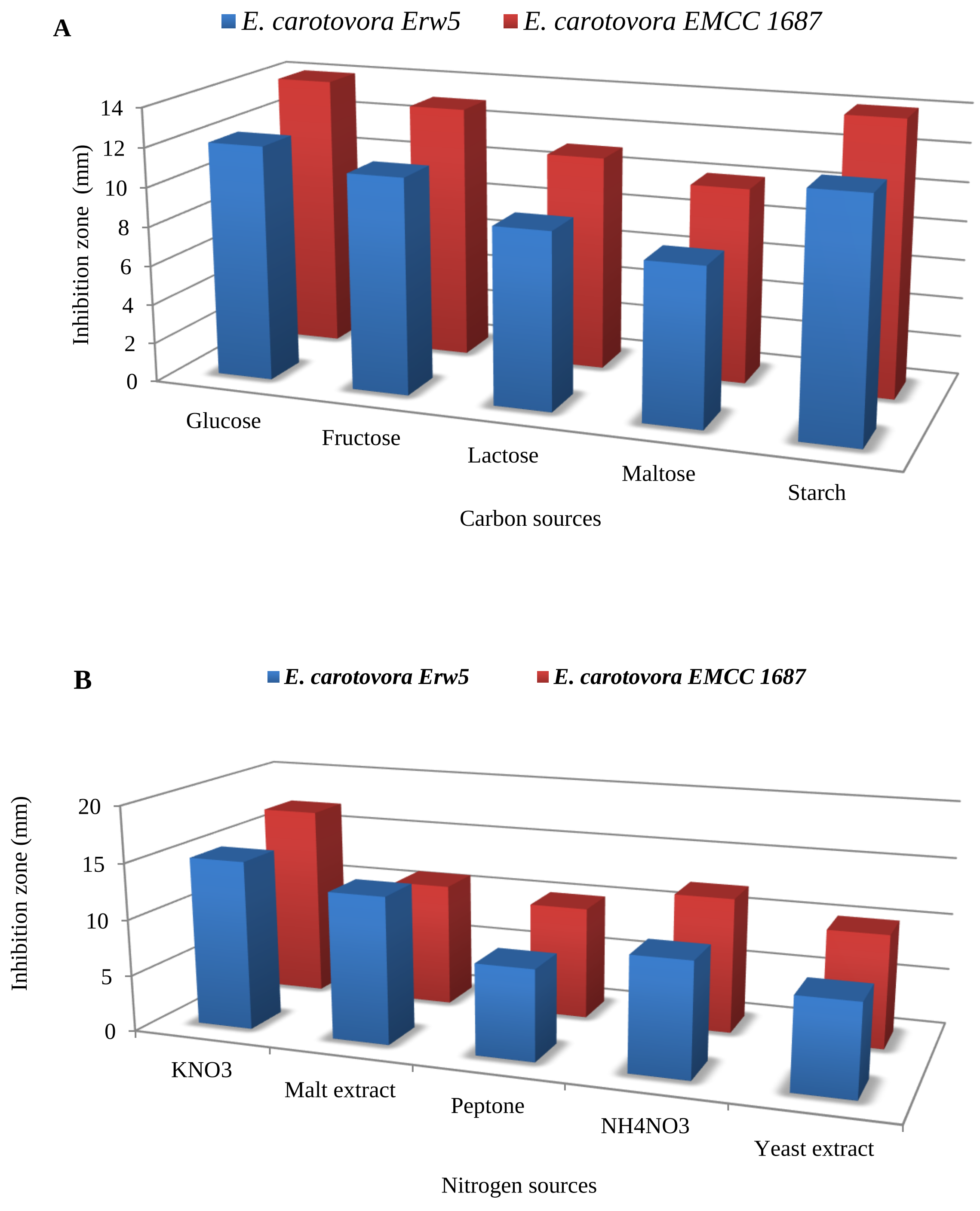

Figure S2: Effect of carbon and nitrogen sources at different temperature on the antibacterial activity from *S. californics* (22/30a) against of *E. carotovora* Erw$_5$ and *E. carotovora* EMCC 1687; A: carbon sources; B: nitrogen sources.

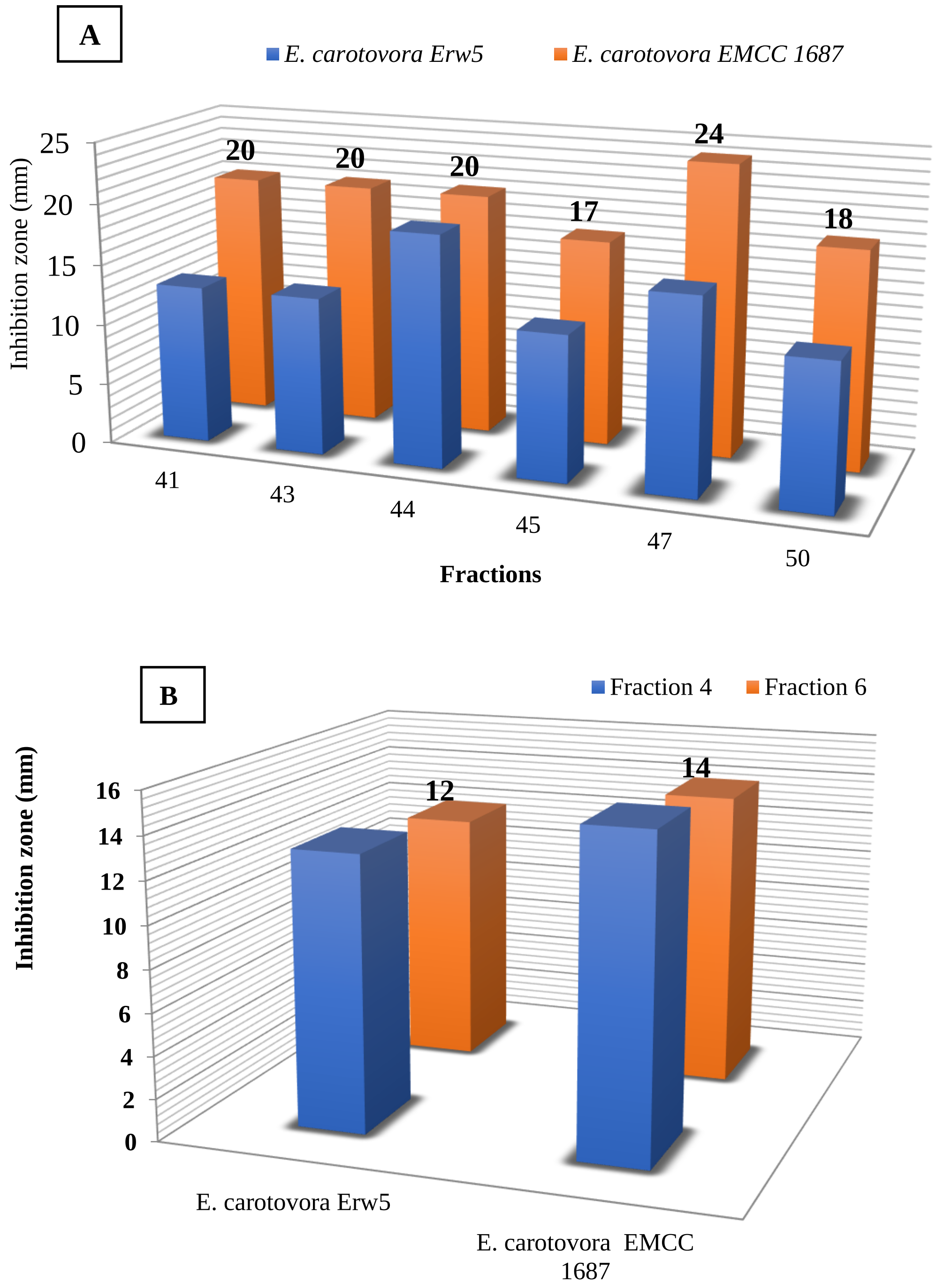

Figure S3: Assay of antibacterial activity of fractions of *S. californics* (22/30a) against *E. carotovora* Erw_5 and *E. carotovora* EMCC 1687. A: fraction 41-50; B: *E.* fraction 4 and 6.

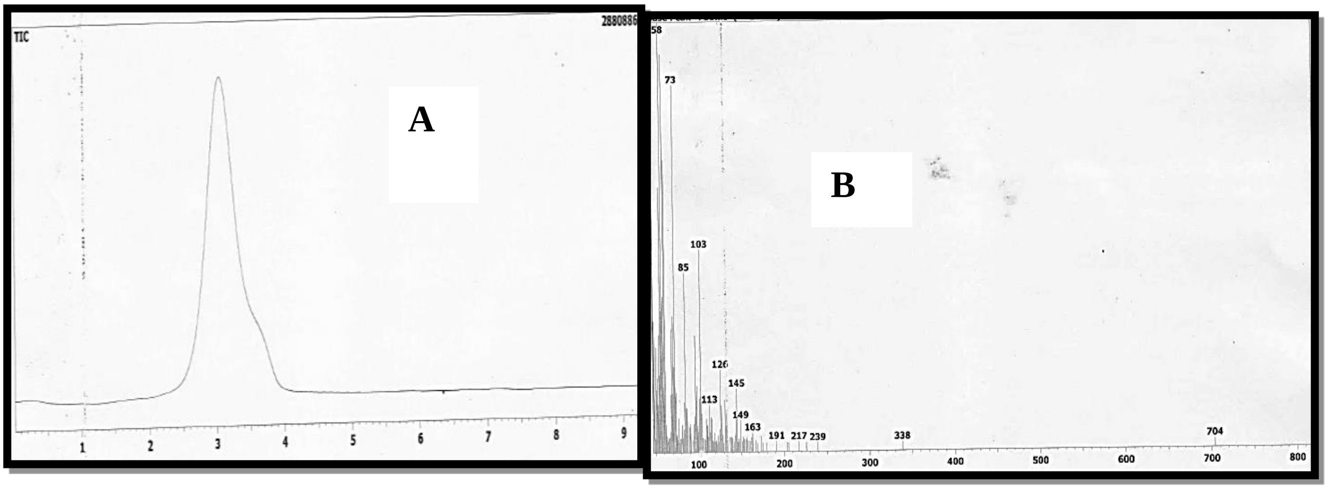

Figure S4: GC Chromatogram of fraction No. 6 (A), and Mass Spectrum of antibacterial agent fraction No. 6 [Ret. Time: 3.433] (B).
